# A comparison of survival outcomes and side effects of toremifene or tamoxifen therapy in premenopausal estrogen and progesterone receptor positive breast cancer patients: a retrospective cohort study

**DOI:** 10.1186/1471-2407-12-161

**Published:** 2012-05-01

**Authors:** Ran Gu, Weijuan Jia, Yunjie Zeng, Nanyan Rao, Yue Hu, Shunrong Li, Jiannan Wu, Liang Jin, Lijuan Chen, Meijun Long, Kai Chen, Lili Chen, Qiaozhen Xiao, Mei Wu, Erwei Song, Fengxi Su

**Affiliations:** 1 Department of Breast Surgery, Sun Yat-Sen Memorial Hospital, Sun Yat-Sen University, Guangzhou, 510260, China; 2 Department of Pathology, Sun Yat-Sen Memorial Hospital, Sun Yat-Sen University, Guangzhou, 510260, China; 3 Republic Health, Sun Yat-Sen University, Guangzhou, 510000, China

**Keywords:** Tamoxifen, Toremifene, Breast cancer, Adjuvant endocrine therapy, Premenopausal

## Abstract

**Background:**

In premenopausal women, endocrine adjuvant therapy for breast cancer primarily consists of tamoxifen alone or with ovarian suppressive strategies. Toremifene is a chlorinated derivative of tamoxifen, but with a superior risk-benefit profile. In this retrospective study, we sought to establish the role of toremifene as an endocrine therapy for premenopausal patients with estrogen and/or progesterone receptor positive breast cancer besides tamoxifen.

**Methods:**

Patients with early invasive breast cancer were selected from the breast tumor registries at the Sun Yat-Sen Memorial Hospital (China). Premenopausal patients with endocrine responsive breast cancer who underwent standard therapy and adjuvant therapy with toremifene or tamoxifen were considered eligible. Patients with breast sarcoma, carcinosarcoma, concurrent contralateral primary breast cancer, or with distant metastases at diagnosis, or those who had not undergone surgery and endocrine therapy were ineligible. Overall survival and recurrence-free survival were the primary outcomes measured. Toxicity data was also collected and compared between the two groups.

**Results:**

Of the 810 patients reviewed, 452 patients were analyzed in the study: 240 received tamoxifen and 212 received toremifene. The median and mean follow up times were 50.8 and 57.3 months, respectively. Toremifene and tamoxifen yielded similar overall survival values, with 5-year overall survival rates of 100% and 98.4%, respectively (*p* = 0.087). However, recurrence-free survival was significantly better in the toremifene group than in the tamoxifen group (*p* = 0.022). Multivariate analysis showed that recurrence-free survival improved independently with toremifene (HR = 0.385, 95% CI = 0.154-0.961; *p* = 0.041). Toxicity was similar in the two treatment groups with no women experiencing severe complications, other than hot flashes, which was more frequent in the toremifene patients (*p* = 0.049). No patients developed endometrial cancer.

**Conclusion:**

Toremifene may be a valid and safe alternative to tamoxifen in premenopausal women with endocrine-responsive breast cancer.

## Background

The optimal adjuvant therapy for premenopausal women with hormone-responsive breast cancer still remains unclear despite the many clinical trials over the past 60 years that have attempted to address this issue. Historically, tamoxifen has been overlooked for the treatment of breast cancer in premenopausal women, due to the false belief that it is ineffective in this patient group. Indeed, it was not until 1995 that the Early Breast Cancer Trialists' Collaborative Group review unequivocally rejected this misconception by demonstrating its efficacy in lowering the rate of recurrence and mortality in premenopausal women with HR-positive breast cancer [1]. In recent years, the impact of endocrine therapies – primarily consisting of tamoxifen and ovarian suppression strategies – as an adjuvant treatment for premenopausal patients with early breast cancer is well established. Moreover, the expert panel of the 2009 St Gallen Conference accepted tamoxifen (or tamoxifen plus ovarian suppression) as the gold standard in endocrine therapy for this group [2].

Although there is mounting evidence to show that tamoxifen has the potential to serve as adjuvant endocrine therapy for women of all ages with breast cancer, questions on the long-term safety of tamoxifen have been raised, due to the adverse event associated with this therapy, including the increased risk of secondary endometrial cancers, the formation of pulmonary embolism, deep vein thrombosis and stroke [3-7]. More rarely, hepatotoxicity, ocular problems and an increased risk of colorectal cancer have also been reported [8-16].

Recently, several alternative hormonal adjuvant therapies for the treatment of breast cancer have become available. Of these, toremifene is a synthetic analogue of tamoxifen and, like tamoxifen, acts on the estrogen receptor. Although, the clinical efficacy of toremifene and tamoxifen are comparable, both as palliative and postmenopausal adjuvant therapies [17-23], toremifene and tamoxifen are metabolized differently, due to a single side chain chloride ion, leading to a more favorable toxicity profile [24-27]. Data on secondary endometrial cancer showed that the incidence of this cancer was lower with toremifene than tamoxifen, prompting speculation that toremifene may unmask existing endometrial tumors rather than induce new events. Further, the risk of stroke, pulmonary embolism, and cataract may be lower with toremifene than with tamoxifen. Other evidence suggests that the beneficial estrogen agonist effects of toremifene, including changes in bone mineral density and lipid profiles, were equivalent to those of tamoxifen [28].

A prospective study using MRI demonstrated that uterine changes associated with adjuvant drugs for breast cancer occur exclusively in postmenopausal patients receiving selective estrogen receptor modulators [29]; however, those studies mostly focused on postmenopausal patients. For premenopausal women receiving tamoxifen, menopausal symptoms seemed to be a significant concern, although the side effects of tamoxifen alone were milder, with the exception of vaginal discharge [30]. Thus, the gynecological side-effects of endocrine therapy differ according to menstrual status. This may be related to the action of tamoxifen on the human endometrium in postmenopausal women, where it has simple estrogenic effects including hyperplasia, whereas in premenopausal women it is linked with endometrial cystic atrophy. Moreover, postmenopausal patients treated with tamoxifen may develop endometriosis, adenomyosis and leiomyomata. Tamoxifen also disrupts the menstrual cycle and increases the incidence of ovarian cysts in premenopausal breast cancer patients while in postmenopausal patients it induces ovarian cystic tumors and endometriomas [31,32]. In addition, tamoxifen causes a decrease in bone mineral density in premenopausal women [33,34]. However, in postmenopausal women, only a mild bone-sparing effect during breast cancer therapy has been reported, suggesting that in these women, anti-estrogens may act as estrogen agonists in the bone [35,36]. Taken together, such findings prompt the question: as an adjuvant endocrine therapy for premenopausal breast cancer patients, is toremifene superior to tamoxifen?

The use of toremifene as an adjuvant endocrine therapy for premenopausal patients with breast cancer is rarely reported. Although one can point to a study on 632 patients aged under 50 receiving toremifene as an adjuvant therapy, this report was aimed at tamoxifen therapy in very young breast cancer patients, and it did not compare the different effects of toremifene and tamoxifen, and did not evaluate the duration of treatment [37]. In view of these limitations, the purpose of this study was to compare these SERMs in premenopausal estrogen or progesterone receptor positive women and planned to perform a safety analysis, using a retrospective and cohort clinical study.

## Methods

### Patients

Patients with early invasive breast cancer who were treated between January 1998 and June 2009, were selected by searching breast tumor registries at the Sun Yat-Sen Memorial Hospital. Premenopausal patients with endocrine responsive breast cancer who underwent standard therapy and adjuvant TAM/TOR for 5 years were eligible for inclusion in our study. Their premenopausal status was confirmed by measuring E2 and FSH levels before surgery (in accordance with the menopausal criterion of the 2010 National Comprehensive Cancer Network). Patients were excluded if they had breast sarcoma, carcinosarcoma, or concurrent contralateral primary breast cancer, had distant metastasis at diagnosis, or had not undergone surgery in combination with endocrine therapy.

### Procedures

In our study, all patients had unifocal, stage I–III invasive breast cancer and had received local control in the form of modified radical mastectomy (plus radiotherapy if more than 3 lymph nodes were involved) and breast-conserving surgery followed by radiotherapy. Chemotherapy and antiestrogen therapy were administered in accordance with 2005 St Gallen treatment guidelines (Table 1 and Table 2). Patients at an intermediate or high risk of recurrence received six to eight cycles of chemotherapy at 21-day intervals using one of the following regimens: cyclophosphamide 500 mg/m^2^, methotrexate 50 mg/m^2^, 5-fluorouracil 500 mg/m^2^ (CMF), epirubicin 90 mg/m^2^, docetaxel 75 mg/m^2^ or paclitaxel 175 mg/m^2^ (ET); 5-fluorouracil 500 mg/m^2^, epirubicin 90 mg/m^2^, cyclophosphamide 500 mg/m^2^ (FEC); docetaxel 75 mg/m^2^ or paclitaxel 175 mg/m^2^, cyclophosphamide 500 mg/m^2^ (TC). In our department, antiestrogen therapy was offered following chemotherapy for all patients with endocrine responsive tumors regardless of their risk category, whereby endocrine responsiveness was defined as the presence of a ER and/or PR positive status regardless expression level [38]. Past research has shown that daily toremifene (60 mg) is as effective as daily tamoxifen (20 mg) in the treatment of postmenopausal hormone-dependent breast cancer, hence in our study, 20 mg of tamoxifen and 60 mg of toremifene were given daily, orally, for 5 years [39]. Besides those listed above, no other concomitant therapy was used, except for medication for conditions unrelated to the breast cancer. One doctor prescribed patients tamoxifen routinely, and another doctor toremifene. 

**Table 1:** Definition of risk categories for breast cancer patients who had undergone surgery, according to the 2005 St. Gallen guidelines

**Risk Category**	**Patient Features**
**Low risk**	**Node negative AND all of the following features:**
	pT ≤2 cm,
	Grade 1,
	Absence of peritumoral vascular invasion,
	HER2/neu gene neither overexpressed nor amplified,
	Age ≥35 years
**Intermediate risk**	**Node negative AND at least one of the following features:**
	pT >2 cm,
	Grade 2–3,
	Presence of peritumoral vascular invasion,
	HER2/neu gene overexpressed or amplified,
	Age >35 years
	**Node positive (1–3 involved nodes) AND**
	HER2/neu gene neither overexpressed nor amplified
**High risk**	**Node positive (1–3 involved nodes) AND**
	HER2/neu gene overexpressed or amplified
	**Node positive (4 or more involved nodes)**

**Table 2:** Differences between treatment modalities as per the 2005 St. Gallen guidelines

**Risk Category**	**Endocrine responsive**	**Endocrine response uncertain**	**Endocrine nonresponsive**
**Low risk**	ET or Nil	ET or Nil	Not applicable
**Intermediate risk**	ET alone, or	CT → ET	CT
	CT → ET	(CT + ET)	
	(CT + ET)		
**High risk**	CT → ET	CT → ET	CT
	(CT + ET)	(CT + ET)	

Abbreviations: ET, endocrine therapy; Nil, no adjuvant systemic therapy; CT, chemotherapy.

The primary outcome measured was overall survival, defined as the time from surgery to death from any cause. The secondary endpoint was recurrence-free survival, which was defined as the time from surgery to first evidence of recurrent disease (consisting of local recurrence and distant metastasis) or death from breast cancer. Patients known to be alive without recurrent disease or lost to follow-up at the time of analysis were screened at the time of their last follow-up.

The study was also designed to collect data on the toxicity of the endocrine therapy. In this context, the patients were interviewed over the telephone or asked to fill out a questionnaire. At each visit, data was collected on the following: nausea, presence of hot flashes, sweating, vaginal bleeding, vaginal dryness, leukorrhea, diarrhea, changes in mood or depression, the presence of skin rashes or itching, and whether the patient had experienced any symptoms or history of arterial or venous thromboses. The results of other tests including ultrasound/CT and blood test results were also collected by searching the patients’ medical records. Follow-up was performed until Feb 2011 and included: breast–abdomen ultrasound, chest X-ray every 3 months within the first 2 years after surgery, breast–abdomen–transvaginal ultrasound, and chest X-ray every 6 months within 3–5 years. Yearly mammograms, breast MR (if necessary), bone scans, chest-abdomen CT and brain MR were also performed, as was the evaluation of a number of biochemical and histological markers.

### Ethics

The study was conducted in accordance with the current revision of the Declaration of Helsinki of the World Medical Assembly, and in conformity with regulations concerning clinical trials issued by medical authority at the Sun Yat-Sen Memorial Hospital (No. 2,2010). The study protocol and all relevant amendments were reviewed and approved by Ethics Committees of the Sun Yat-Sen Memorial Hospital.

### Statistical methods

Statistical analyses were performed using SPSS 13.0 for Windows (SPSS Inc., Chicago IL,USA). Correlation was assessed using a χ² test and survival rates were estimated using a Kaplan–Meier test. A log-rank test was used to compare survival curves and cox regression analysis was used to balance the risk factors for prognosis between groups. The prognostic factors included in the multivariate analysis model were: patient age, tumor size, lymph node status, histological grade, Her-2 status, chemotherapy, type of adjuvant endocrine therapy drug, and local control. A *p* value of less than 0.05 was considered to be significant.

## Results

**Figure 1:**
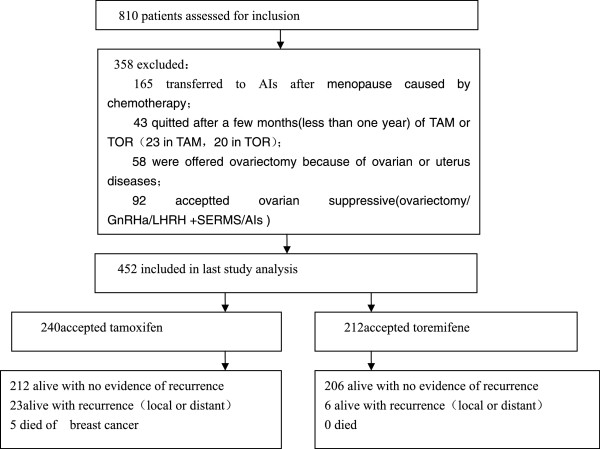
**Schematic illustrating the overall study design.** Survival outcomes, recurrence-free outcomes and the presence of side effects in tamoxifen or toremifene treated premenopausal breast cancer patients were evaluated.

Patients in our study all presented with self-detected breast cancer. Figure 1 shows the number of patients assessed at every stage in the study. A total of 810 patients with breast cancer were reviewed, of whom 452 were eligible for inclusion in the study. The median follow up time was 50.8 months, the mean follow up time was 57.3 months (range: 17.3 - 209 months, SD 29.5 months). The clinical and pathological characteristics of eligible patients are shown in Table 3. The median age of the group was 43 years (IQR 38.0 - 46.8), and mean age was 41.8 ± 5.9 years. No significant difference in the distribution of these variables was noted, except for the type of local control. Similarly, no significant difference was observed between the two treatment groups of patients who died of breast cancer (*p* = 0.138).

**Table 3:** Patient characteristics characterized according to whether the patients received tamoxifen (TAM) or toremifene (TOR) treatment

	**TAM (n = 240)**	**TOR (n = 212)**	**P value**
**Age**			0.552
18-34	34	26	
>35	206	186	
**T**			0.744
1	147	137	
2	84	67	
3	9	8	
**N**			0.536
0	142	136	
1-3	61	52	
4-9	27	19	
≥10	10	5	
**AJCC Stage**			0.616
I	98	97	
IIA	70	64	
IIB	32	25	
IIIA	30	21	
IIIB	0	0	
IIIC	10	5	
**HER-2 status**			0.359
Positive	37	23	
Negative	148	137	
Unknown	55	52	
**Grade**			0.328
I	51	37	
II	141	120	
III	47	52	0.530
Unknown	1	3	
**Risk of recurrence**			0.313
Low	11	15	
Intermediate or	229	197	
high			0.024
**Local control**	117	130	
BCS + RT	90	57	
Modified	33	25	
Modified + RT			0.513
**Chemotherapy**	15	10	
CMF	96	92	
ET	62	42	
FEC	43	45	
TC	24	23	
No chemotherapy			

Differences between the two groups were evaluated using a χ^2^ test.

**Figure 2:**
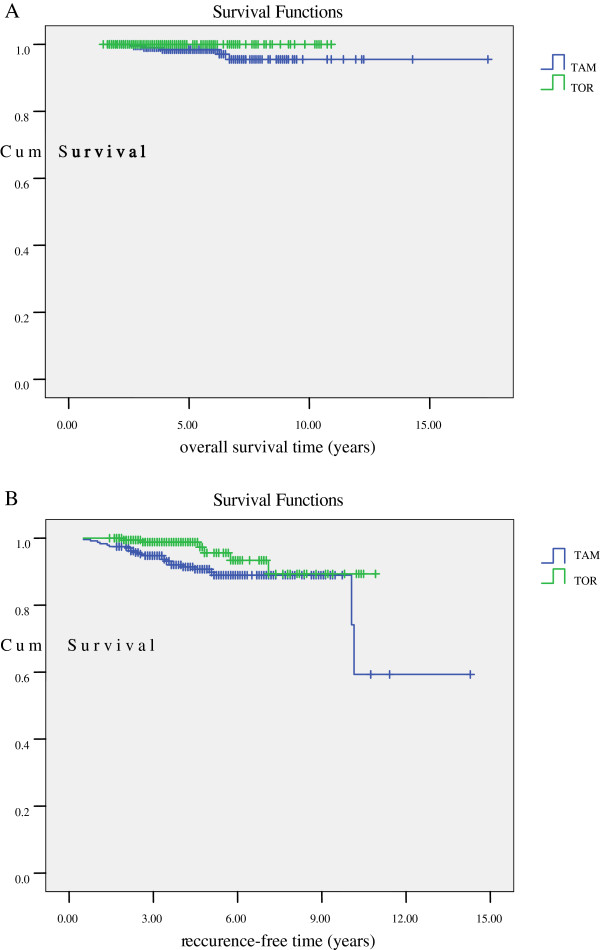
**A. Kaplan–Meier analysis was used to evaluate overall survival in tamoxifen (TAM) or toremifene (TOR) treated premenopausal breast cancer patients (*p*** **= 0.087).** B. Kaplan–Meier analysis was used to evaluate recurrence-free survival in tamoxifen (TAM) or toremifene (TOR) treated premenopausal breast cancer patients (*p* = 0.022).

Cox regression analysis for all patients included in the study showed that recurrence-free survival in the toremifene group was significantly longer than in the tamoxifen group (HR = 0.385, 95% CI = 0.154-0.961; *p* = 0.041; Table 4). However, multivariate analysis confirmed that no factor was independently related to overall survival (Table 5). We did further Kaplan–Meier analysis of survival with patients in the two treatment groups (Figure 2, Table 6). For overall survival (OS), the 3-year, 5-year and 8-year OS was better in the toremifene patients, who were all alive at the time of last follow up. However, stratified log-rank tests for unadjusted analyses, did not reveal a significant difference. For recurrence-free survival, survival outcomes were improved following toremifene treatment compared with tamoxifen (*p* = 0.022).

**Table 4:** Multivariate analysis of recurrence-free survival between patients treated with tamoxifen (TAM) and toremifene (TOR)

	**HR (95%CI)**	**P value**
Age	0.960(0.903-1.020)	0.191
T	1.602(0.844-3.041)	0.150
Grade	0.817(0.547-1.220)	0.323
N	1.543(0.998-2.385)	0.051
Her-2	0.950(0.856-1.055)	0.336
Local control	0.933(0.521-1.672)	0.816
Chemotherapy	1.249(0.898-1.739)	0.187
TAM-TOR	0.385(0.154-0.961)	0.041

**Table 5:** Multivariate analysis of overall survival between tamoxifen (TAM) and toremifene (TOR) treated patients

	**HR(95%CI)**	**P value**
Age	1.012(0.859-1.192)	0.887
T	0.873(0.156-4.873)	0.877
Grade	1.025(0.376-2.793)	0.962
N	1.656(0.586-4.678)	0.341
Her-2	1.096(0.891-1.347)	0.387
Local control	0.630(0.150-2.643)	0.528
Chemotherapy	0.993(0.441-2.234)	0.987
TAM-TOR	0.000(0.000-6E + 174)	0.955

**Table 6:** Comparison of recurrence and death rates in premenopausal patients with breast cancer treated with tamoxifen (TAM) and toremifene (TOR)

	**TAM (n = 240)**	**TOR (n = 212)**	**P value**
Overall survival(%)	97.9	100	0.087
3 years	99.5	100	
5 years	98.4	100	
8 years	95.5	100	
Recurrence-free survival(%)	90.4	97.2	0.022
3 years	94.8	98.8	
5 years	90.7	95.6	
8 years	88.9	89.3	

Differences between the groups were evaluated using log-rank tests for unadjusted analyses.

Of the patients enrolled in this study, 29 (6.4%) experienced breast cancer recurrence during the mean follow-up period of 50.2 months. Of these patients, 23 (9.6%) of those taking tamoxifen and 6 (2.8%) of those taking toremifene had a local, regional, distant, or multi-site recurrence (Table 7). In the tamoxifen group, there were 12 patients who had multi-site recurrence, of which 7 had more than one site of distant metastases, 5 who had concurrent locoregional and one or more sites of distant recurrence. In the toremifene group, there was 1 patient who had concomitant bone and liver metastases. We noted a higher number of local recurrences as well as distant recurrences in women taking tamoxifen; however, the differences between the two groups were not statistically significant. Five (1.1%) of the women died during the study period, all of whom had been offered tamoxifen (2.1%; Table 7). Of those 5 women, all of the deaths were attributed to breast cancer.

**Table 7:** Site of recurrence and the cause of death in premenopausal breast cancer patients treated with tamoxifen or toremifene

	**Tamoxifen (n = 240)**	**Toremifene (n = 212)**
Recurrences	23(9.6%)	6(2.8%) 2
Logoregional	14	
Distant	15	4
Lymph nodes or other soft tissues	3	2
Bone	8	2
Liver	4	1
Contralateral breast cancer	1	0
Brain	2	0
Lung	4	0
Deaths	5(2.1%)	0(0%)
Breast cancer	4	0
Other reasons	1	0

In the tamoxifen group, there were 12 patients with multiple site of recurrence, of which 7 had more than one site where distant metastases were detected, 5 had concurrent locoregional and one or more sites of distant recurrence. In the toremifene group, there was 1 patient who had concomitant bone and liver metastases.

Our study shows a trend toward similar rates of subjective and objective events in the women treated with tamoxifen or toremifene (Table 8). No women experienced severe adverse events such as thromboembolic or cerebrovascular complications. Hot flashes were the only side effect, which when compared between the two groups was statistically significant. There were 90 patients in tamoxifen group and 76 patients in toremifene group who underwent menopause during endocrine therapy (*p* = 0.716), and some of them turned to aromatase inhibitors (AIs; Table 9, *p* = 0.242). Table 9 also lists the common reasons for discontinuing the drug regimen besides death or recurrence, and shows that patient compliance was similar in the two groups.

**Table 8:** Side effects and adverse events in premenopausal breast cancer patients treated with tamoxifen or toremifene

	**Tamoxifen**	**Toremifene**	**p value**
**Subjective**			
Sweating	59	63	0.220
Hot flash	56	67	0.049
Vaginal discharge	12	7	0.369
Leucorrhea increasing	22	31	0.072
Vaginal dryness	18	13	0.566
Vaginal bleeding	3	4	0.869
Skin pruritus	19	18	0.824
Depression	17	22	0.213
Skinrash	15	13	0.959
Nausea or vomit	3	8	0.082
Diarrhea	3	3	1.000
Asomnia	22	25	0.361
Weightgain	50	45	0.918
Fracture	2	1	1.000
Ostalgia	49	49	0.488
Hyper-menorrhea	2	7	0.124
Blurred vision	5	3	0.857
**Objectives**			
Ovarian cysts	12	18	0.137
Teratoma	1	1	1.000
Uterine fibroids	25	35	0.057
Hysterectomy	9	17	0.052
Uterine polyps	2	3	0.889
Cervical cyst	2	0	0.501
Endometrial hyperplasia	15	22	0.110
Osteoporosis	4	2	0.796
Fatty liver	25	20	0.728
Hemangiomas	4	3	1.000
Hepatic cyst	8	5	0.536
Cholecystic polips	3	4	0.869
Transaminase step-up	2	7	0.426
Splenic hemangioma	0	1	0.469
Cataracts	0	1	0.469

**Table 9:** Adherence to therapy in premenopausal breast cancer patients treated with tamoxifen or toremifene

	**Tamoxifen (n = 240)**	**Toremifene (n = 212)**	**p value**
Total	45	31	0.242
Transfer to AIs after menopause	24	15	0.269
Discontinuance or other therapy	21	16	0.542
Cervical cyst	1	0	
Uterine fibroids	1	0	
Ostalgia	1	0	
Skinrash	2	0	
Endometrial hyperplasia	5	0	
In order to be pregnancy	1	1	
Diarrhea	1	0	
To fear adverse effect	1	4	
Economic reason	1	0	
Transaminase step-up	1	2	
Chinese traditional medicine	2	0	
Discontinuance for unknow reasons	4	6	
Hyper-menorrhea	0	2	
Vaginal bleeding	0	1	

## Discussion

Results of this retrospective analysis suggest that toremifene and tamoxifen have similar efficacies in premenopausal breast cancer patients, and have comparable side effects. Although the standard therapy for premenopausal women with breast cancer is tamoxifen, some patients are offered treatment with ovarian suppression in conjunction with AI therapy, either because tamoxifen is contraindicated or they have an intolerance to tamoxifen, or because their physicians believe that the AIs are superior based on data from postmenopausal women. Although AIs are increasingly being used in breast cancer patients, the importance of classic drugs for premenopausal patients should not be ignored. Chemotherapy in young breast cancer patients frequently causes abrupt menopause, although many menstruate again following chemotherapy, especially very young patients. Further, some postmenopausal patients recommence menstruation after treatment with AIs. As is widely known, AIs are harmful for premenopausal patients. However, with respect to toremifene clinical trials have shown that toremifene does not increase the incidence of adverse events for moderate to severe mastalgia patients [40]. Other reports have shown that toremifene (60 mg daily) has no substantial negative effects on bone mineral density in pre- or postmenopausal women and may actually have a minor favorable influence [41]. In this situation, when considering safety tamoxifen or toremifene should be considered. However, few studies have investigated the therapeutic role of toremifene as adjuvant endocrine therapy for premenopausal breast cancer patients. Since there are more young breast cancer patients in Asian countries, than in other regions, researchers from Asia are increasingly becoming interested in this issue. In a Korean study, toremifene was suggested to young patients, but this study failed to compare the impact and side effect of tamoxifen and toremifene, and the treatment duration was not followed up [37]. Another study, from the Seoul National University College of Medicine, evaluated the use of toremifene as an adjuvant hormonal therapy for estrogen receptor positive early breast cancer patients in terms of its therapeutic efficacy and effect on the endometrium, as compared with tamoxifen, but the study did not consider how treatment affected these patients’ menstrual cycles. In our study, therapy duration was investigated, in addition to the effect of treatment on the menstrual cycle, by evaluating estradiol and follicle stimulating hormone levels.

Multivariate analysis showed that toremifene was associated with improved recurrence-free survival and that no factor was independently related to overall survival. Future studies may need to increase samples sizes in order to establish the optimal therapy. Moreover, our study was also limited by a lack of investigation as the expression of the estrogen and progesterone receptor and changes in HER-2 status.

All objective adverse events were obtained by searching the internal hospital database and medical records during the period when the patients were undergoing endocrine therapy. With respect to the gynecological side-effect such as ovarian cysts and uterine fibroids, these always occurred a few months after taking the drugs, and were mostly so mild and stable that we did not need to disrupt the endocrine therapy. As for other side effects, such as skin rash and nausea, the patients were frequently unable to recall when these events had occurred, the seriousness, or the possible causes.

In our study, most patients were treated concurrently, and following an almost identical protocol of surgical intervention plus chemotherapy, but with differing adjuvant endocrine therapies. There was no difference in age, tumor size, tumor grade, degree of lymph node involvement, or risk of recurrence between the two cohorts. Wherever possible we attempted breast-conserving surgery unless there were positive margin, in which case radical mastectomy was performed. As the women who received breast-conserving surgery always had a significantly lower tumor load, other factors should also considered, such as the tumor-breast ratio and tumor location. Similarly, as BCS is the marriage of breast conserving surgery and radiation therapy, the side effects of radiotherapy should not be ignored. In China, breast-conserving surgery costs are more expensive than modified radical mastectomy because of the surgery costs, concomitant radiotherapy, and the additional pathology required. These factors were explained to each of the patients, and the type of surgery was then based on input from both patients’ and doctors’. In spite of the issues around cost and side effects and in light of the traditional bias towards breast-conserving surgery, some patients elected for radical mastectomy.

Inclusion of only one center in our study may be related to the clustering of surgeries between the two cohorts, which may explain why there was a nearly 13% difference in local treatment modality between the two groups and possibly reflects the fact that doctors influenced patients during the talk before surgery. Among young women, those who received BCS are more likely to experience local recurrence, while long-term survival was similar for those who received BCS compared to those who underwent modified radical mastectomy [42]. In our study, BCS was higher in the toremifene group but the risk of recurrence was significantly lower. The study also benefited from a large patient population, with few lost to follow-up, and a long follow-up period (median >50 months).

Compliance with treatment was suboptimal in both arms of the study, with 23 women on tamoxifen 20 women on toremifene ceasing therapy after a few months (*p* = 0.961; Figure 1). Many turned to traditional Chinese medicine and refused any further consultation. These patients were not included in the final analysis. However, patients who discontinued tamoxifen and toremifene within 5 years because of menopause or other unavoidable reasons were still included, in accordance with intent-to-treat analysis.

## Conclusions

This work represents the first study comparing the clinical efficacy and side effects of tamoxifen and toremifene in premenopausal breast cancer patients. The results demonstrated that toremifene has similar effect as tamoxifen, but can prevent further recurrence. These findings are important for clinicians treating premenopausal breast cancer patients who are beginning adjuvant endocrine therapy.

## Competing interests

The authors confirm that they have no competing interests to declare.

## Authors’ contributions

Ran Gu, Weijuan Jia, Yunjie Zeng, Nanyan Rao were the principal investigators of the study and they contributed equally to the study design and manuscript preparation. Ran Gu, Weijuan Jia, Yunjie Zeng, Yue Hu, Shunrong Li, Jiannan Wu, Liang Jin, Meijun Long, Kai Chen, Chen Lili, Qiaozhen Xiao, Mei Wu contributed to the data collection. Yunjie Zeng reviewed the pathology. Ran Gu and Lijuan Chen performed the data analysis and interpretation. Ran Gu,Weijuan Jia and Nanyan Rao drafted the Tables and Figures. Erwei Song and Fengxi Su are guarantors for the study.

## Pre-publication history

The pre-publication history for this paper can be accessed here:

http://www.biomedcentral.com/1471-2407/12/161/prepub
